# State-of-the-art application of nanoparticles in radiotherapy: a platform for synergistic effects in cancer treatment

**DOI:** 10.1007/s00066-024-02301-y

**Published:** 2024-10-04

**Authors:** Mehrnaz Mostafavi, Farhood Ghazi, Mahboobeh Mehrabifard, Vahid Alivirdiloo, Mobasher Hajiabbasi, Fatemeh Rahimi, Ahmad Mobed, Gholamreza Taheripak, Marzieh Ramezani Farani, Yun Suk Huh, Salar Bakhtiyari, Iraj Alipourfard

**Affiliations:** 1 Faculty of Allied Medicine, Shahid Beheshti University of Medical Sciences, Tehran, Iran; 2 Faculty of Medicine, Tabriz University of Medical Science, Tabriz, Iran; 3 Hormozgan University of Medical Sciences, Bandarabas, Iran; 4 Ramsar Campus, Mazandaran University of Medical Sciences, Ramsar, Iran; 5 Islamic Azad University of Tonekabon, Tonekabon, Iran; 6 Division of Clinical Laboratory, Zahra Mardani Azar Children Training Research and Treatment Center, Tabriz University of Medical Sciences, Tabriz, Iran; 7 Social Determinants of Health Research Center, Tabriz University of Medical Sciences, Tabriz, Iran; 8 Department of Biochemistry, School of Medicine, Iran University of Medical Sciences, Tehran, Iran; 9 NanoBio High-Tech Materials Research Center, Department of Biological Sciences and Bioengineering, Inha University, Incheon, Korea (Republic of); 10 Department of Clinical Biochemistry, School of Medicine, Ilam University of Medical Sciences, Ilam, Iran; 11 Iraj Alipourfard, Institute of Physical Chemistry, Polish Academy of Sciences, Marcina Kasprzaka 44/52, 01-224 Warsaw, Poland

**Keywords:** Radiotherapy (RT), Nanoparticles (NPs), Glioblastoma (GBS), Prostate cancer (PCa), Breast cancer

## Abstract

Radiotherapy (RT) is a gold standard cancer treatment worldwide. However, RT has limitations and many side effects. Nanoparticles (NPs) have exclusive properties that allow them to be used in cancer therapy. Consequently, the combination of NP and RT opens up a new frontier in cancer treatment. Among NPs, gold nanoparticles (GNPs) are the most extensively studied and are considered ideal radiosensitizers for radiotherapy due to their unique physicochemical properties and high X‑ray absorption. This review analyzes the various roles of NPs as radiosensitizers in radiotherapy of glioblastoma (GBS), prostate cancer, and breast cancer and summarizes recent advances. Furthermore, the underlying mechanisms of NP radiosensitization, including physical, chemical, and biological mechanisms, are discussed, which may provide new directions for next-generation GNP optimization and clinical transformation.

**Graphic abstract:**

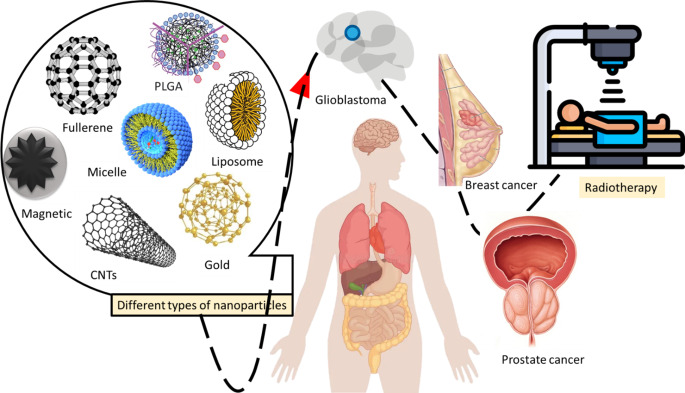

## Introduction

Cancer is a major threat to global public health. It is a major cause of significant morbidity and mortality worldwide each year [1, 2]. Approximately 1,918,030 cancer cases and 609,360 cancer-related deaths were reported in 2022, and 26 million new cases of cancer are expected by 2030 [2]. Cancer arises from the unrestrained proliferation of cells due to pathophysiological changes in the inherent cell division process and subsequently spreads to various tissues [3]. Surgery and radiotherapy play important roles in the treatment of primary, nonmetastatic solid tumors. However, combination chemotherapy is common in patients with comorbidities and deep-seated tumors, especially those involving large blood vessels or brain tumors, who are not candidates for surgery [4]. Radiotherapy, including internal radioisotope therapy and external-beam radiation therapy, plays an important role in the treatment of metastatic tumors, early and late-stage solid tumors, and regional lymph nodes. This type of treatment depends on cellular damage that occurs when living tissue is exposed to ionizing radiation [5, 6]. To improve radiotherapy sensitization, several products have been developed and used in clinical practice, including radiosensitizers such as glycididazole sodium and injectable irisquinone capsules [7]. Additionally, the combination of radiotherapy and anticancer drugs has produced a synergistic effect in tumor treatment, for example through the use of cisplatin and paclitaxel [8]. However, the challenge is that currently used chemotherapy and radiotherapy both cause significant cytotoxicity in normal tissues, and free chemotherapeutic agents rarely accumulate at the tumor site [7, 8]. The development of new radiosensitization strategies that can increase the efficacy of radiotherapy while reducing side effects remains a major challenge in efforts to improve radiotherapy outcomes [7, 8]. Nanoparticles offer unique opportunities for radiotherapy due to their high surface area-to-volume ratio, improved cellular uptake, and ease of functionalization [9]. Nanoparticles are composed of high‑Z elements and can act as radiosensitizers to external ionizing radiation beams or be used as carriers to deliver therapeutic radionuclides. By functionalizing the nanoparticle surface with targeting molecules, the delivery of tumor-specific therapeutic doses can be achieved [10, 11]. Alternatively, in passive targeting, tumor accumulation can be achieved by coating the target with nanoparticles and controlling the circulation time of the nanostructures [10, 11]. In this review, we summarize some important discoveries regarding radiotherapy using newly developed gold nanoparticles and highlight some of the mechanisms discovered and the methods developed. This review study has attracted significant attention from diverse communities and provides both experimental and computational insights for studies from the molecular to the cellular level. The overview is structured as follows: first, we show the main mechanisms of radiation therapy using photons and ions. Next, the mechanism of achieving radiosensitization using nanoparticles (NPs) is presented, followed by the influence of different physicochemical properties (size, material, coating, charge) of such NPs, and their toxicity and biodistribution effects are then discussed. Finally, the paper concludes with an overview of the field and future challenges.

## Conventional radiotherapy

**Fig. 1:**
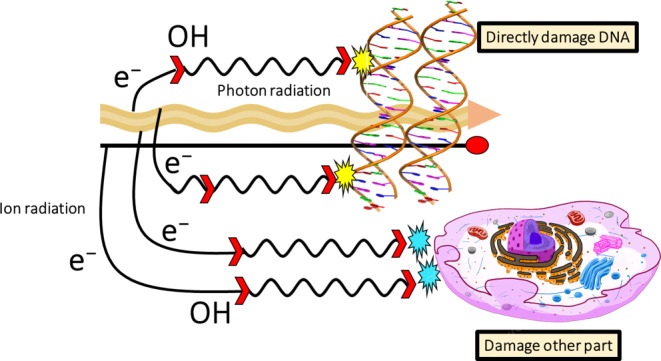
Illustration of the mechanisms of radiation damage. In the case of the direct effect, IR directly damages the DNA, which, if unrepaired, results in cell death or permanent growth arrest. In the case of the indirect effect, ROS are formed by the radiolysis of a large amount of water and oxygen, and the ROS then damage the DNA. There are many types of DNA damage, such as base change, SSB, DSB, and cross-linkage with protein or with other DNA molecules. (Adapted from [12]). *IR* ion radiation, *ROS* reactive oxygen species, *SSB* single strand break, *DSB* double strand break

Radiotherapy, which delivers ionizing radiation locally through external beams or surgical implantation of radionuclide-based seeds into the tumor, is one of the gold standard treatments for cancer. Because radiation is nonselective, healthy tissue surrounding the cancerous area is usually affected by the treatment. After irradiation of biological media, various events occur on different timescales. These events are commonly referred to as physical, chemical, and biological steps. Although damage to parts of the cell other than DNA, such as mitochondrial damage, can also lead to cell death, the most commonly used indicator to determine the effectiveness of radiation therapy is the number of DNA strand breaks created (Fig. 1).

Both photon and ion radiation can damage DNA and other parts of cells. Mitochondria directly damage and ionize the medium, producing radicals and other reactive species (represented here as ⋅OH radicals) as well as secondary electrons that can cause indirect damage after diffusion. Secondary electrons can also react with the medium to increase the number of radicals [12]. Advances in technology over the past few decades have allowed for better and more precise radiation delivery to the target. Conventional radiation therapy (CONV) typically uses fractionated doses of 2 Gy per day for 5 days over several weeks [13]. The distinction between tumor and normal tissue responses is achieved through dose fractionation, a method that exploits the tissue-sparing effect by allowing healthy tissues more time to recover between radiation doses, minimizing damage to normal cells while targeting tumor cells effectively[13]. However, when treating radioresistant tumors, such as high-grade brain or pancreatic malignancies, the total radiation dose that can be delivered to the tumor is often limited by the sensitivity of adjacent critical normal tissues, which prevents complete treatment due to their vulnerability to damage [13]. In such situations, ultra-high-dose rate FLASH RT may be of great interest. FLASH-RT strategies are typically considered to involve delivering radiation doses to the target volume at dose rates of 40 Gy/s or higher, whereas CONV uses dose rates of 0.01 to 0.1 Gy/s [14, 15]. Due to the different irradiation times, CONV irradiation occurs during chemical and biological reactions, whereas FLASH does not interact with these biochemical steps [14, 15].

**Fig. 2:**
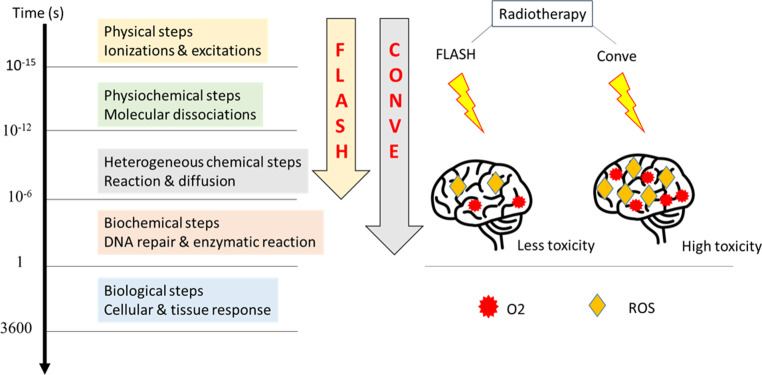
Differential physicochemical events distinguish FLASH from CONV irradiation. (Adapted from [14]). *ROS* reactive oxygen species

As revealed in Fig. 2, FLASH irradiation is 1000 times faster than CONV irradiation when delivering similar doses. CONV irradiation occurs during ongoing chemical and biological reactions, but FLASH does not interact with these initial radiation reactions. FLASH induces rapid oxygen deprivation and transient local hypoxia, reducing reactive oxygen species (ROS) levels and normal brain toxicity compared to CONV irradiation. The three primary types of ROS include the superoxide anion (O_2−_), hydrogen peroxide (H_2_O_2_), and the hydroxyl radical (HO). The most common ROS is O_2_, which is converted by mitochondrial SOD into H_2_O_2_ [14]. The ultimate goal of RT is to destroy all diseased cells while leaving healthy tissue behind. RT has made significant progress toward this goal over the past few decades. This is partly due to new technologies such as improved computer-aided inverse treatment planning, multileaf collimator-assisted modulation of radiation beams, image guidance, stereotactic therapy, precision robotics, better motion-management strategies, and hypofractionation techniques. With recent developments in nanotechnology, targeted RT using gold nanoparticles (GNPs) is being actively investigated to increase the therapeutic rate of RT.

## Advanced radiotherapy

Nanotechnology, which offers many unique properties for oncology applications, has also proven to be a promising strategy to improve the efficacy of radiotherapy [16, 17]. By leveraging the enhanced permeability and retention (EPR) effect: (A) contrast enhancement in image-guided radiotherapy (RT) can be achieved, (B) preferential accumulation of nanoparticles within tumors can be improved, and (C) combined chemotherapeutic and radiotherapeutic strategies can be optimized [18, 19]. It may lead to tumor-specific delivery of therapeutic agents. Moreover, NPs lead to an increased local radiation dose by using particles with a higher atomic number (Z) [20, 21]. Among the various nanoplatforms investigated for radiotherapy applications, gold nanoparticles (AuNPs) are known for their high X‑ray absorption coefficients and precise control over the physicochemical properties of the particles. These are the most extensively studied due to the ease of synthetic manipulation [18, 19]. Spatially fractionated radiation therapy (SFRT) is a radiotherapy method that hypothetically allows safe dose escalation for large tumors. Special beam collimation produces high-dose peaks distributed throughout the target volume and low-dose valleys in between [22, 23]. Fractionated radiation therapy can deliver a higher dose per fraction to the target tumor and induce immune activation (modulation of TME). Therefore, combining NPs with fractionated radiation therapy may significantly improve the prognosis of metastatic cancer [22]. Considering the importance of using nanoparticles for enhancing radiotherapy, the next part of the article is dedicated to radiotherapy based on nanoparticles in the treatment of some cancers. To increase the scientific richness, the discussed studies include the most recent published articles in this field.

## Nanoparticle-based radiotherapy

### Prostate cancer

Prostate cancer (PCa) is the second most common malignancy in men globally and the fifth leading cause of cancer-related death, with 1.4 million new cases and 370,000 deaths anually [24, 25]. It is estimated that 5000 people have died from metastatic castration-resistant prostate cancer (PCs), despite initial treatment success in earlier stages of the disease. This highlights the urgent need for more effective therapies to combat the advanced stages of PCs [24, 25]. The significant mortality and morbidity associated with disease progression is primarily due to the lack of specific and sensitive PCa screening systems, detection of the disease at advanced stages, and failure of cancer treatment [24, 25]. In response to the development of radiopharmaceuticals targeting prostate-specific membrane antigen (PSMA), nanotechnology-based delivery systems with various payloads are being explored for potential use in PCa-targeted imaging, treatment, and therapeutic applications [26, 27]. Among these, NPs labeled with radionuclides are attracting increasing attention for use in nuclear medicine applications. However, the success of targeted cancer therapy and imaging using radiolabeled NPs depends on the selection of an appropriate radiolabeling strategy, the type of NP, the type of radionuclide, the type of ligand (targeting vector), and the stability of the radiolabeled complex [26, 27]. As a result of a huge number of studies, today, a large and diverse number of nanoparticles are available for potential use in PCa-targeted nuclear nanomedicine, the most important of which include [28] iron oxide NPs, quantum dots (QDs), gold NPs, texaphyrin NPs, polymer NPs, micellar NPs, melanin NPs, liposomal NPs, copper sulfide NPs, gadolinium vanadate NPs, and zeolite NPs [28–31]. The conjugation of Gd(III) complexes with PSMA-targeting ligands to AuNP surfaces leads to enhanced uptake of AuNPs by PSMA-expressing cancer cells, with excellent MRI contrast and radiation therapy outcomes in vivo and in vitro. The findings revealed prostate cancer-targeted AuNPs for MRI-guided radiotherapy to increase targeting precision and efficacy [32]. A novel platform was developed using tetragonal ultrathin nanosheets (NSs) of 64 Cu-labeled GdVO4:4% Eu, a two-dimensional (2D) material that simultaneously exhibits fluorescence, radioactivity and paramagnetic properties. This allows for multimodal imaging of prostate cancer (PC), enabling more accurate diagnosis and monitoring. The carboxyl-functionalized Eu^3+^-doped GdVO_4_ NSs were synthesized by a facile solvothermal reaction followed by ligand exchange with polyacrylic acid (PAA) [33]. Targeted lipid nanoparticles (LNPs) showed a twofold increase in tumor uptake compared to single-chain antibody fragments (scFvs) alone when using two different thiol ester chemistries. Anti-prostatic membrane antigen (PSMA) scFv-LNPs showed a 1.6-fold increase in tumor targeting compared to nontargeting LNPs. In other words, targeted anti-PSMA scFv LNPs showed increased tumor accumulation compared to scFv alone or nontargeted DOTA micelles, providing evidence for the development of this drug delivery system [34]. CuS NPs were synthesized by Na_2_S and ^64^CuCl_2_ reaction in aqueous solution. Then, PEG linkers with or without bombesin peptides were conjugated to the surface of [^64^Cu]CuS NPs to yield PEG-[^64^Cu]CuS NPs and Bom-PEG-[^64^Cu]CuS NPs. These two kinds of NPs were used to test specific uptake in prostate cancer cells in vitro and for imaging of orthotopic prostate tumors in vivo [35]. A ^68^Ga-magnetic iron oxide nanoparticle (mNP) targeting gastrin-releasing peptide (GRPR) and PSMA receptors is a potential device for the diagnosis of PCa with PET/MRI [36]. An innovative radioimmunoconjugate, ^223^RaA-silane-PEG-D2B, was fabricated for targeted mCRPC therapy. The original compound involves the α‑particle-emitting ^223^Ra radionuclide with NaA zeolite nanocarrier loaded functionalized by the PSMA D2B antibody [37]. Organic melanin nanoparticles were applied to PSMA small molecular groups, and the lengthy half-life radionuclide ^124^I (t_1/2_ = 100.8 h) was straight labeled. The developed platforms were efficiently bonded to the surface of NPs to make the PSMA-targeted long-retention nanoprobe ^124^I‑PPMN, which can potentially increase tumor uptake and prolong residence time. The created system could be used as a safety device for the precise imaging of prostate cancer with high expression of PSMA due to the application of an elemental radionuclide with labeled organic melanin nanoparticles [38]. The biodistribution and pharmacokinetics of the PSMA-targeted nanoparticle based on a poly(lactic acid)-polyethylene glycol copolymer was investigated by fluorescence imaging of a low-molecular-weight, single-photon-emission computed tomography (SPECT) and PSMA-targeting moiety attached to the surface and oriented toward the outside environment [39]. A liposome-based system was developed for theranostic delivery to (PSMA^+^) LNCaP cells. A lipopolymer (P^3^) involving polyethylene glycol (PEG_2000_), PSMA ligand (PSMAL), and palmitate was produced and post-inserted into the surface of preformed liposomes. These P^3^ liposomes were loaded with radiolabeled ^99m^Tc radionuclide and doxorubicin to investigate their theranostic characteristics [28]. A theranostic nanotexaphyrin was advanced for focal photodynamic therapy (PDT) and PSMA-targeted radionuclide imaging accomplished through the chelation of metal isotopes (In, Lu). In this work, a robust and rapid ^111^In/Lu-nanotexaphyrin radiolabeling technique using a microfluidic system that achieved a high radiochemical yield was fabricated to realize nanotexaphyrin’s theranostic properties [40]. ^89^Zr-PEG-(DFB)_1_(ACUPA)_3_, ^89^Zr-PEG-(DFB)_3_ (ACUPA)_1_, and prostate cancer-targeting starPEG nanocarriers (40 kDa, 15 nm) with one or three PSMA-targeting ACUPA ligands were designed and synthesized. Results showed that an increased number of ACUPA ligands enhanced the in vitro binding affinity of PEG-derived polymers to PC3-PIP cells([30]; Table 1).

**Table 1:** Developed nanoparticle-based radiotherapy for prostate cancer

NP	NP size	Radionuclide	Final compound	Ligand	Platform	Ref.
AuNPs	7.8 nm	3 H-labeled ZJ24	Au-Gd(III)-PSMA NPs	Cys-PSMA‑1	PET	[32]
Gadolinium vanadate NPs (GdVO_4_)	∼150 nm	Copper-64	^64^Cu-DOTA-GdVO 4:4% Eu-DGEA	Asp-Gly-Ala (DGEA) peptide	PET/MR	[33]
Micellar NPs (LNP)	12 nm	Copper-64	^64^Cu-DOTA-scFv-LNP	Single chain (scFv)	PET	[34]
Copper sulfide NPs (CuS)	5 nm	Copper-64	Bom-PEG-[^64^Cu]CuS	Bombesin (7–14)	PET	[35]
Iron oxide NPs (mNP-S1/2; mNP-N1/2)	55–138 nm	Gallium-68	^68^Ga-mNP-N1/2 ^68^Ga-mNP-S1/2	Glutamate-urea-lysine ligand bombesin (7–14)	PET/MRI	[36]
Zeolite NPs	~120 nm	Radium-223	^223^RaA-silane-PEG-D2B	Antibody D2B	Therapy	[37]
Melanin NPs (MNPs)	13 nm	Iodine-124	^124^I‑MNPs-PEG-TL	Glutamate-urea-lysine ligand	PET	[38]
Poly(lactic acid)-polyethylene glycol NPs (PLA-PEG)	~100 nm	Indium-111	^111^In-DOTA-RDye680RD-PEGPLA-ACUPA RDye680RD-PEG-PLA-ACUPA	ACUPA	SPECT/NIFR	[39]
Doxorubicin-containing liposomal NPs modified with P3-liposomes	~180 nm	Technetium99m	^99^mTc-P3-liposomes	Glutamate-urea-lysine ligand	Theranostic SPECT/chemotherapy	[28]
Texaphyrin NPs (texaphyrin)	~100 nm	Indium-111 Lutetium-175	^111^In/175Lu-texaphyrin-TL	Glutamate-urea-lysine ligand	Theranostic SPECT/NIRF/PDT therapy	[40]
Polymer PEG NPs (PEG-(DFB)1; PEG-(DFB)3)	15 nm	Zirconium-89	^89^Zr-PEG-(DFB)3(ACUPA)1 ^89^Zr-PEG-(DFB)1(ACUPA)3	ACUPA	PET	[30]
NP∓PVP + B + Pt	_	Bismuth and cisplatin	NP∓PVP + RT (VI)	DNA	X‑ray	[41]
Folate-polyethylenimine 600-cyclodextrin	140 nm	_	Dbait∓H1 NPs	_	XR	[42]

*NP* nanoparticle, *AuNPs* Gold Nanoparticles, *PSMA* Prostate-Specific Membrane Antigen, *PET* Positron Emission Tomography, *MR* Magnetic Resonance Imaging, *DOTA* 1,4,7,10-Tetraazacyclododecane-1,4,7,10-tetraacetic acid (chelator for radiolabeling), *DGEA* Aspartic Acid-Glycine-Glutamic Acid-Alanine (targeting peptide), *scFv* Single-Chain Variable Fragment (antibody fragment), *PEG* Polyethylene Glycol, *SPECT* Single-Photon Emission Computed Tomography, *NIRF* Near-Infrared Fluorescence Imaging, *PDT* Photodynamic Therapy, *Dbait DNA* Double-Strand Break Repair Inhibitor, *XR* X-Ray, *TL* Targeting Ligand, *RT* Radiotherapy

### Glioblastoma

**Fig. 3:**
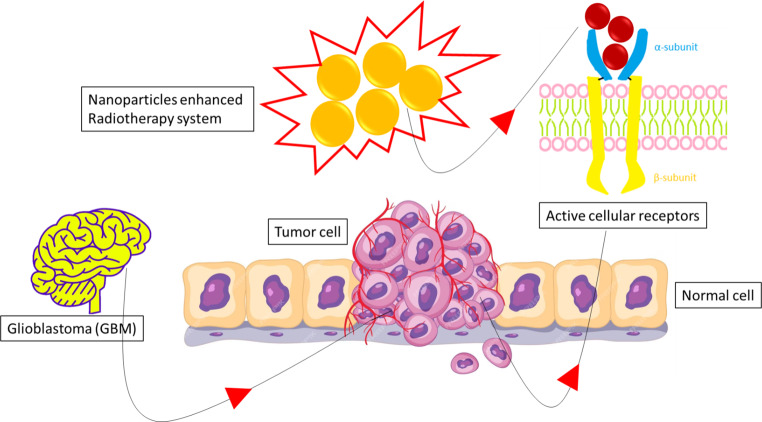
Schematic illustration of a nanoparticle-enhanced radiotherapy system. (Adapted from [43])

Glioblastoma (GBM), also known as grade IV astrocytoma, is a rapidly growing, aggressive brain tumor [43, 44]. It invades nearby brain tissue but usually does not spread to distant organs. GBM is the most common and devastating primary brain tumor, with an age-adjusted incidence of 3.23 per 100,000 people and an average annual incidence of 12,000 cases, accounting for 57.7% of all gliomas and 48.6% of all malignant brain tumors [43, 44]. Despite extensive efforts to improve outcomes, the current standard of care remains limited, as demonstrated by a median overall survival (OS) of 14.6 to 16 months and a 5-year survival rate of 4.6% [43, 44]. Active tumor targeting occurs not only by reaching tumor cells but also by localizing nanocarriers in specific intracellular organelles or spaces such as the cell nucleus or mitochondria [45, 46]. Moreover, active targeting can mean reaching the tumor area, targeting the tumor microenvironment, targeting angiogenesis in the tumor microenvironment, or directly targeting tumor cells by internalization, taking into account the route of administration [45, 46]. Active targeting involves the use of carriers with various surface ligands to achieve transport across the intact blood–brain barrier (BBB) or cellular uptake after extravasation across the leaky BBB [45, 46]. A schematic illustration of a nanoparticle-enhanced radiotherapy system is presented in Fig. 3.

In recent years, various studies have been developed for the treatment of glioblastoma based on radiotherapy nanoparticles. For example, the hypoxic radiosensitization effects of a novel hypoxia-responsive angioprep-2-lipid-poly(MIs)n (ALP-(MIs)n) polyprodrug nanoparticle (NP) for targeted glioma therapy [47]. The findings indicated that the Fe_3_O_4_-Au-C225 composite targeted MNPs have the potential to be used as a tracer for glioma in vivo [48]. Radiosensitizing Fe-Au nanocapsules (Hybridosomes) improved survival of GL261 brain-tumor-bearing mice treated by radiotherapy [49]. In novel research, magnetic resonance imaging (MRI)-guided focused ultrasound with cisplatin-conjugated GNP (Cis-UP-GNP) was fabricated for GB treatment. This treatment inhibited glioma cells by inducing DNA damage and apoptosis both in vivo (NOD SCID gamma mice submitted to xenotransplantation of U251 GB cells) and in vitro (U251 and U87 GB cell lines) when compared to free cisplatin. Also, a synergistic effect with radiotherapy was revealed [50]. The results of one study showed a hydrodynamic diameter of 192 nm with doxorubicin-carrying hollow superparamagnetic iron oxide NPs (SPIONs; DOX-SPION) for the treatment of U87 cells in GBM [51]. Solid lipid nanoparticles (SLNs) conjugated to a cyclic peptide iRGD (CCRGDKGPDC) deliver small interfering RNAs (siRNAs) against both PD-L1 and epidermal growth factor receptor (EGFR) for targeted and immunotherapy against GBM [52]. Nanoparticle-based radiotherapy was developed for the treatment of recurrent glioblastoma multiforme. The results show that the magnetic fluid (MFL) can be dispersed in very small portions (tens of milliliters) over the target area, producing an accurate specific absorption rate (SAR) distribution, which provides a thermal control of therapy that can be greatly improved [53]. Iron oxide nanoparticles, in combination with intracavitary hyperthermia and radiotherapy, have been developed as local treatments for recurrent GBM. The developed system could induce a significant inflammatory response around the resection cavity and induce a strong antitumor immune response, which could lead to long-term stabilization of patients with recurrent GBM [54]. As mentioned above, the combination of nanoparticles and radiotherapy may represent a promising treatment for GBM patients at first relapse, but initial results justify the need for further research (Table 2).

**Table 2:** Developed nanoparticle-based radiotherapy for glioblastoma (GBM)

NP	NP size	Radionuclide	Final compound	Ligand	Platform	Ref.
MNPs	30-nm	^T224^h post-MNP	Poly(d,l-lactic-*co*-glycolic acid)-*b*-polyethylene glycol (PLGA-PEG)	_	_	[47]
MNPs	35 nm	^_^	Fe_3_O_4_-Au-C225	α (TGF-α)	MK3-353 model	[48]
Fe-AuNPs	_	Hafnium	Fe-Au nanocapsules (Hybridosomes)	GBM cells	MRI	[49]
GNPs	7 nm	Phalloidin-594	Cis-UP-GNP	_	MRgFUS	[50]
SPIONs	192 nm	39.6 Gy	SPIONs-Fe_3_O_4_	EGFRvIII	MRI	[51]
SLN	50.2 nm	6‑Diamidino-2-phenylindole (DAPI)	6‑Diamidino-2-phenylindole (DAPI)-SLN	EGFR and ligand‑1 (PD-L1)	_	[52]
MNPs	15 nm	16–70 Gy	–	–	MRI	[53]
SPIONs	12 nm	5 × 1.8 Gy per week	SPIONs-Fe_3_O_4_	_	MRI	[54]
Gold NPs	20 nm	^177^Lu-Au	Lu-AuNLS-RGD-anti-VEGF aptamer	RGD	XR	[55]
AuNPs	_	Iodine, gadolinium	IGd (AuNPs)	_	MRI	[56]

*NP* nanoparticle, *MNPs* Magnetic Nanoparticles, *Fe-AuNPs* Iron-Gold Nanoparticles, *GNPs* Gold Nanoparticles, *SPIONs* Superparamagnetic Iron Oxide Nanoparticles, *SLN* Superparamagnetic Lipid Nanoparticles, *T224h post-MNP* Time 224 hours after Magnetic Nanoparticles treatment, *PLGA-PEG* Poly(d,l-lactic-co-glycolic acid)-b-polyethylene glycol, *Fe3O4@-Au-C225* Iron Oxide-Gold-C225 (a type of antibody), *α (TGF-α)* Alpha Transforming Growth Factor Alpha, *MK3-353 model* A specific model used for, *testingHybridosomes* A proprietary term for Fe-Au nanocapsules, *GBM cells* Glioblastoma Multiforme Cells, *MRI* Magnetic Resonance Imaging, *Phalloidin-594* A fluorescent dye used for labeling, *Cis@-UP@-GNP* A compound involving cisplatin and gold nanoparticles, *MRgFUS* Magnetic Resonance-guided Focused Ultrasound, *39.6 Gy*: A measure of radiation dose (Gray units), *EGFRvIII* Epidermal Growth Factor Receptor variant III, *DAPI* 6-diamidino-2-phenylindole, a fluorescent stain, 6*-diamidino-2-phenylindole (DAPI)-SLN*: DAPI-labeled Superparamagnetic Lipid Nanoparticles, *EGFR* and *Ligand-1 (PD-L1)* Epidermal Growth Factor Receptor and Programmed Death Ligand 1, *Lu–AuNLS-RGD-antiVEGF aptamer* Lutetium-Au nanoparticle conjugated with a peptide targeting RGD and an anti-VEGF aptamer, *RGD* Arginine-Glycine-Aspartic acid peptide, *XR* X-ray imaging, *IGd (AuNPs)* Iodine-Gadolinium conjugated Gold Nanoparticles

### Breast cancer

**Fig. 4:**
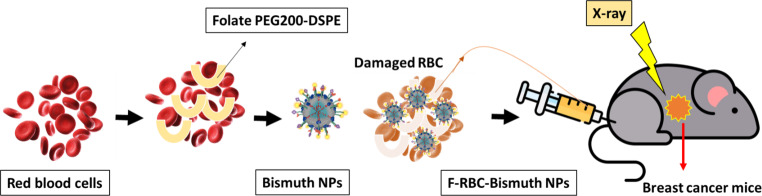
Tumor-targeted bismuth nanoparticles enhanced by X‑ray irradiation for breast cancer. (Adapted from [59]). *RBC* red blood cell, *F-RBC-Bismuth NPs* folate red blood cell bismuth nanoparticles

Gold nanoparticles (GNPs) have been proposed in combination with radiotherapy to improve tumor control. However, the exact mechanism underlying GNP radiosensitization is still not understood. Therefore, a new method to estimate the increase in radiosensitivity caused by nanoparticles was developed. In this study, the model using in vitro survival data of MDA-MB-231 breast cancer cells that received different concentrations of 2‑nm diameter GNPs and different doses of 160-kVp, 6‑MV, and 15-MV photons were validated [57]. Internalization of AuNPs through dose escalation was used as a radiosensitizer for rotational radiotherapy of breast cancer using kilovoltage (kV) X‑ray beams. No effect on cell viability was demonstrated by AuNPs alone. Cell viability curves after irradiation with doses up to 2 Gy showed increased mortality with AuNPs compared to irradiation without AuNPs [58]. One study showed that the interaction of bismuth nanoparticles and X-rays not only destroys cancer cells but also dissolves nanoparticles in stable physiological conditions (Fig. 4). This research found that bismuth nanoparticles, modified with a red blood cell membrane and conjugated with folate, were used in X-ray radiation therapy for breast cancer. In this study, folate acts as a tumor-targeting agent, and cell membrane coating provides a long circulation time [59].

Iodine absorbs X‑rays during radiotherapy (RT), generates free radicals and local tumor damage, and effectively increases the local RT dose to the tumor. This study shows that in radiation therapy trials, some animal models using iodine nanoparticles (INPs) + RT have shown impressive lifespan extensions of up to 10 times or more compared to RT alone [60]. An original study proposed using HER2-targeting ^177^Lu (trastuzumab-AuNP-^177^Lu) and nontargeting AuNP-^177^Lu for killing and tumor inhibition of HER2-overexpressing breast cancer (BC) cells in vitro and proceeded to evaluate the efficacy of trastuzumab-modified AuNPs and compare in vivo proliferation after intratumoral injection [61]. The potential synergy of RT with immunostimulatory chitosan/poly(γ-glutamate) nanoparticles (Ch/γ-PGA-NPs) in inducing antitumor immunity in orthotopic breast tumor 4T1 in a mouse model was confirmed. Results showed that untreated animals exhibited progressive primary tumor growth and developed splenomegaly and lung metastases. RT reduced primary tumor burden, whereas treatment with Ch/γ-PGA NPs reduced systemic immunosuppression and lung metastasis ([62]; Table 3).

**Table 3:** Developed nanoparticle-based radiotherapy for breast cancer

NP	NP size	Radionuclide	Final compound	Ligand	Platform	Ref.
GNPs	2 nm	Radiobiological LEM	LEM-GNPs	MDA-MB-231 breast cancer cells	–	[57]
GNPs	15 nm	_	GNPs-MDA-MB-231 cells	MDA-MB-231 cells	kV-ebRT	[58]
Bismuth	40 nm	X‑ray	F‑RBC bismuth NPs	Lipid-tethered targeting ligands	X‑ray radiotherapy	[59]
INPs	20 nm	X‑rays	(INPs) + RT	TNBC, MDA-MB-231 cells	X‑ray radiotherapy	[60]
AuNP	1.5 nM	^177^Lu	^177^Lu (trastuzumab-AuNP-^177^Lu) targeted to HER2	HER2	Trastuzumab-AuNP-^177^Lu to SK-BR-3 cells	[61]
Ch/γ-PGA NPs	1.4 nM	X‑rays	RT + Ch/γ-PGA NPs	MDSCs	X‑ray radiotherapy	[62]
Au NPs	17 and 41 nm	X‑rays	Gd_2_O_3_-BSA-Au NPs	Red blood cells (RBCs)	X‑ray radiotherapy	[63]

*NPs* Nanoparticles, *GNPs* Gold Nanoparticles, *LEM* Low-Energy Microscopy, *LEM-GNPs* Gold Nanoparticles modified with Low-Energy Microscopy, *kV-ebRT* Kilovolt electron beam Radiation Therapy, *Bismuth* Bismuth Nanoparticles, *F-RBC* Folate-conjugated Red Blood Cell membrane, *INPs* Indium Nanoparticles, *TNBC* Triple-Negative Breast Cancer, *HER2* Human Epidermal Growth Factor Receptor 2, *MDSCs* Myeloid-Derived Suppressor Cells, *BSA* Bovine Serum Albumin, *RBCs* Red Blood Cells, *RT* Radiotherapy

Although nanoparticle-enhanced radiotherapy has mostly focused on prostate cancer and glioblastoma, a few studies have been developed on other types of cancers in recent years. In this example, a sulfhydryl cross-linked bacterial cellulose hydrogel (SulBC gel) synthesized by silylation and oxidation reactions was used as a drug delivery system. Next, cis-dichlorodiamine platinum (CDDP) is used as an anticancer agent in a hydrogel (CDDP-SulBC gel) loaded to enable accurate radiotherapy of colorectal cancer. The CDDP-SulBC gel is selectively degraded, resulting in accelerated drug-release behavior in a reducing environment [64, 65]. Gold nanoparticles of dasatinib with anti-HER‑2 aptamer were fabricated for targeted chemoradiotherapy in breast cancer cells. The combination of aptamer and modified dasatinib attached to porous gold nanoparticles with radiation therapy demonstrated an outstanding reduction in the IC50 values for both the MCF‑7 and BT-474 cell lines [66, 67]. The combination of X‑ray-induced radiotherapy and PEGylated selenium nanoparticles (PSNPs) has been used to enhance anticancer efficacy in human lung cancer. Notably, the combined treatment of X‑rays and PSNPs showed predominantly red fluorescence, indicating dead cells. This result demonstrates the cytotoxic potential of the combination of X‑rays and PSNPs against lung cancer cells [68]. Radiotherapy using irinotecan (IRIN) silicasome nanoparticle sensitizes colorectal cancer to immunotherapy by modulating the cGAS/STING pathway. These results suggest that combining radiotherapy with IRIN silicasomes may enhance the effectiveness of immunotherapy through the cGAS/STING signaling pathway, representing a novel strategy for colorectal cancer treatment [69]. Fe_3_O_4_-Au-induced cell damage, leading to apoptosis due to caspase 3/7 activation, secondary necrosis quantified by LDH release, and cell growth arrest as assessed by clone type assay. This study demonstrated the potential of Fe_3_O_4_-Au nanoparticles in enhancing the radiation sensitivity of cancer cells [70].

## Conclusion

Based on the results of some studies, NP-enhanced radiotherapy is only effective with low-energy X‑rays. At the same time, an increase in total dose accumulation is not expected when using particle radiation or high energy, since the interaction between NPs and material is dominated by the Compton effect, which does not strongly depend on Z. However, radiosensitization by high‑Z materials has also been observed for clinical MeV radiation, ion sources, and protons, and physical mechanisms are not exhaustive enough to explain the dose increase caused by the presence of such NPs. The unique properties of nanoparticles enable their use as radiosensitizers after or immediately after surface functionalization with tumor-targeting antibodies. In general, to optimize the therapeutic rate of radiotherapy by nanoparticle radiosensitization, it is necessary to ensure that the distribution of NPs in healthy tissue during radiotherapy is negligible, while tumor cell uptake into tumor cells is important and designed to maximize the accumulation of NPs within. Further studies are needed to investigate various physiological barriers and understand the mechanisms and behavior of NPs in the tumor vasculature and microenvironment.

## Abbreviations

BBB: Blood–brain barrier
BC: Breast cancer
Ch/γ-PGA NPs: Chitosan/poly(γ-glutamic acid) nanoparticles
CONV: Conventional radiation therapy
CuS: Copper sulfide NPs
GBM: Glioblastoma
GdVO_4_: Gadolinium vanadate
GRPR: Gastrin-releasing peptide
INPs: Iodine nanoparticles
kV-ebRT: Kilovolt external-beam radiotherapy
LEM: Local effect model
LNPs: Lipid nanoparticles
MDSCs: Myeloid-derived suppressor cells
MFL: Magnetic fluid
MNPs: Melanin NPs
MRI: Magnetic resonance imaging
NSs: Nanosheets
OS: Overall survival
PDT: Photodynamic therapy
PLA-PEG: Poly(lactic acid)-polyethylene glycol NPs
PSMA: Prostate-specific membrane antigen
ROS: Reactive oxygen species
SAR: Specific absorption rate
scFvs: Single-chain antibody fragments
SLN: Solid lipid nanoparticle
SPECT: Single-photon-emission computed tomography
TNBC: Triple-negative breast cancer
